# Acceptability and operational feasibility of community health worker-led home phototherapy treatment for neonatal hyperbilirubinemia in rural Bangladesh

**DOI:** 10.1186/s12887-024-04584-7

**Published:** 2024-02-15

**Authors:** Farjana Jahan, Sarker Masud Parvez, Mahbubur Rahman, Sk Masum Billah, Farzana Yeasmin, Tania Jahir, Rezaul Hasan, Gary L. Darmstadt, Shams El Arifeen, Md. Mahbubul Hoque, Mohammod Shahidullah, Muhammad Shariful Islam, Sabina Ashrafee, Eric M. Foote

**Affiliations:** 1 Environmental Health and WASH, Health System and Population Studies Division, International Centre for Diarrhoeal Diseases Research, Bangladesh (icddr,b), Dhaka, 1212 Bangladesh; 2 Children’s Health and Environment Program, Child Health Research Centre, The University of Queensland, South Brisbane, QLD Australia; 3 Maternal and Child Health Division, International Centre for Diarrhoeal Diseases Research, Bangladesh (icddr,b), Dhaka, Bangladesh; 4 Faculty of Medicine and Health, School of Public Health, University of Sydney, Sydney, Australia; 5 College of Medicine, Nursing & Health Sciences, National University of Ireland Galway, Galway, Ireland; 6 Prematurity Research Center, Department of Pediatrics, Stanford University School of Medicine, Stanford, California USA; 7 Department of Neonatology, Bangladesh, Children Hospital & Institute, Dhaka, Bangladesh; 8 Bangabandhu Sheikh Mujib Medical University, Dhaka, Bangladesh; 9 National Newborn Health Program (NNHP) and Integrated Management of Childhood Illness (IMCI), Directorate General of Health Services, Dhaka, Bangladesh; 10 Environmental Interventions Unit, Infectious Disease Division, icddr,b, 68, Shaheed Tajuddin Ahmed Sarani, Mohakhali, Dhaka, 1212 Bangladesh

**Keywords:** mHealth, Community health workers, Neonatal health, Neonatal hyperbilirubinemia, Home phototherapy

## Abstract

There is an unmet need for phototherapy treatment in low- and middle-income countries (LMICs) to prevent disability and death of newborns with neonatal hyperbilirubinemia. Home phototherapy deployed by community health workers (CHWs) in LMICs may help increase access to essential newborn postnatal care in a more acceptable way for families and lead to an increase in indicated treatment rates for newborns with hyperbilirubinemia. We aimed to investigate the operational feasibility and acceptability of a CHW-led home phototherapy intervention in a rural sub-district of Bangladesh for families and CHWs where home delivery was common and a treatment facility for neonatal hyperbilirubinemia was often more than two hours from households. We enrolled 23 newborns who were ≥ 2 kg in weight and ≥ 35 weeks gestational age, without clinical danger signs, and met the American Academy of Pediatric treatment criteria for phototherapy for hyperbilirubinemia. We employed a mixed-method investigation to evaluate the feasibility and acceptability of home phototherapy through surveys, in-depth interviews and focus group discussions with CHWs, mothers, and grandparents. Mothers and family members found home phototherapy worked well, saved them money, and was convenient and easy to operate. CHWs found it feasible to deploy home phototherapy and identified hands-on training, mHealth job aids, a manageable workload, and prenatal education as facilitating factors for implementation. Feasibility and acceptability concerns were limited amongst parents and included: a lack of confidence in CHWs’ skills, fear of putting newborn infants in a phototherapy device, and unreliable home power supply. CHW-led home phototherapy was acceptable to families and CHWs in rural Bangladesh. Further investigation should be done to determine the impact of home phototherapy on treatment rates and on preventing morbidity associated with neonatal hyperbilirubinemia. **Clinical Trial (CT) registration ID**: NCT03933423, full protocol can be accessed at https://doi.org/10.1186/s13102-024-00824-6. Name of the trial registry: clinicaltrials.gov. Clinical Trial (CT) registration Date: 01/05/2019.

## Background

An estimated 2.4 million newborns do not have access to phototherapy treatment for hyperbilirubinemia in Central and South Asia [1, 2]. In low-resource settings, where approximately half of births occur at home and access to postnatal care is limited, screening for hyperbilirubinemia and phototherapy treatment is often not available in a timeframe necessary to prevent hyperbilirubinemia-related morbidity and mortality [3, 4]. Of the estimated 481,000 yearly cases of extreme hyperbilirubinemia, 80% occur in low- and middle-income countries (LMICs) [5]. The highest burdens of extreme hyperbilirubinemia and rhesus disease are in sub-Saharan Africa and South Asia, where 74% of the 114,000 yearly deaths occur [5].

Barriers to treatment for families in LMICs include cost, transportation, distance to a hospital, hospitals with inadequate resources to care for newborns with hyperbilirubinemia, loss of work, and lack of housing near hospitals for family members to stay [6, 7]. In addition, there is risk of hospital-acquired infection for newborns in resource-poor settings where newborns with hyperbilirubinemia are treated in close proximity to other newborns with infection [8, 9].

Home phototherapy for hyperbilirubinemia has been found to be effective in some high-income countries when conducted under the supervision of nurses [10–12]. In Bangladesh, several studies showed the effectiveness of a home-based community health worker (CHW) approach in delivering maternal and newborn health interventions including message delivery for health promotion, identification of sick newborns, hospital referral as well as home treatment of newborn sepsis [13–17].

In Bangladesh and other LMICs around the world, CHWs have successfully implemented home-based diabetes management, postnatal newborn care, maternal and child nutrition, and child development interventions [18–28]. CHWs play a crucial role in bridging the gap between community and facility-based services, providing essential maternal and child care at the household level, reducing inequalities in health care for marginalized populations, and improving access [20, 29, 30].

We present here an evaluation of the operational feasibility and acceptability of CHW-led home phototherapy for the treatment of neonatal hyperbilirubinemia in rural Bangladesh using a mixed-methods approach. We examined factors that facilitated treatments as well as barriers experienced by CHWs and families during the implementation of home phototherapy.

## Methods

### Study design and population

This was a cluster randomized controlled trial (NCT03933423) among pregnant women 18 years of age or older in their second or third trimester of pregnancy and the newborns born to enrolled mothers, as described previously [31]. This study was conducted in the Sakhipur sub-district of Tangail district, a rural hard-to-reach area with ~ 300,000 population located 80 km from the capital Dhaka. Around 98% of the pregnant women in the study area have had at least one antenatal care (ANC) visit, 50% of the pregnant women delivered at home, [32] 26% of the mothers delivered via caesarian section, [33] and approximately 62% of the mothers received postnatal care within 2 days of delivery [33]. The nearest hospital for people living in Sakhipur does not have the resources to treat newborns with neonatal hyperbilirubinemia with phototherapy, and newborns are referred to a hospital in the Tangail district 2 h away for treatment.

### Study eligibility

All caregivers and families participating in the intervention arm of the study and whose newborns were eligible for home phototherapy treatment participated in the quantitative evaluation.

### CHW recruitment and training

CHW recruitment and training are detailed in our protocol paper [31]. Briefly, CHWs were female (due to cultural sensitivities with providing breastfeeding support), at least 20 years of age, had completed secondary school, and had a minimum passing score of 60% on a written test of knowledge on community mobilization and sensitization. Previous work experience with government community groups was desirable but not required. Twelve CHWs met the requirements for enrollment were invited for a five-day training, adopted from Darmstadt et al. [34], comprised of theoretical and hands-on training by the physicians. The first phase of training lasted 3 days was theoretical and encompassed neonatal jaundice and responsibilities of CHWs, pregnancy care and maternal danger signs, delivery planning and safe delivery preparations, newborn care, infection prevention practices, breastfeeding and management of breastfeeding problems, risk of developing neonatal jaundice and management, neonatal jaundice symptoms and causes, communication, and counselling. CHW knowledge acquisition was assessed by a pre-test and post-test evaluation [31]. The second phase of training was hands-on and conducted at Dhaka Children’s Hospital over 2-days under the supervision of pediatricians and neonatologists and covered the following topics: recognition of neonatal illness and common neonatal problems, newborn danger sign assessment, postnatal services and the responsibilities of CHWs, referral processes for sick newborns, neonatal jaundice symptoms and causes, management of neonatal jaundice in the home, and training on use of the transcutaneous bilimeter, phototherapy device, and thermometer. CHWs were evaluated with pre-test and post-test assessments on the content of the hands-on training. Three days were also spent by the CHWs performing physical examinations and using equipment in the field site under supervision from the study physician. Ten CHWs that scored the highest on the assessments were invited to participate in the study [31].

#### Components of hands on neonatal jaundice management training

##### Assessment of neonatal danger signs and newborn breastfeeding

**Fig. 1:**
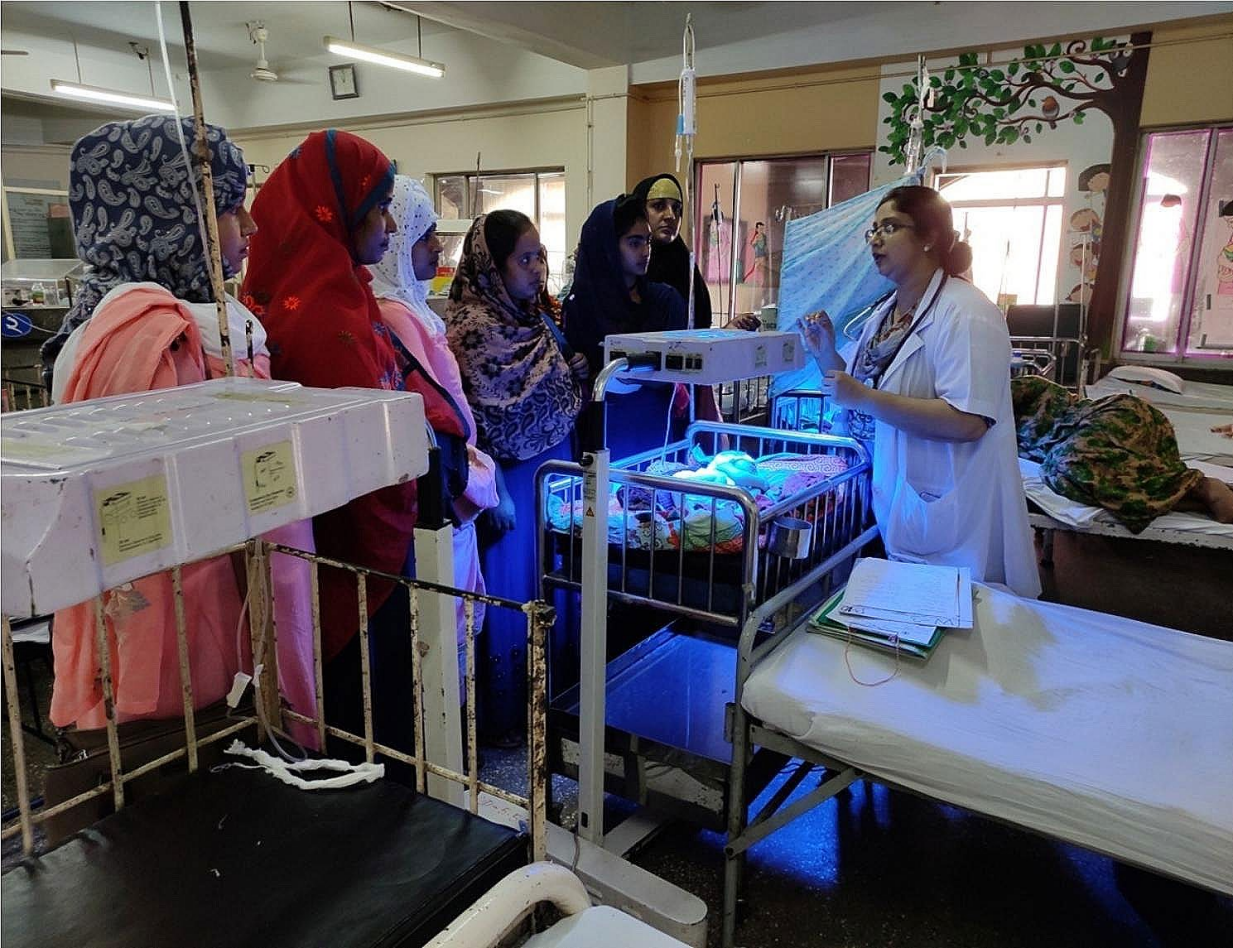
CHWs receiving hands on training on phototherapy administration in Dhaka Children’s Hospital

The hands-on training to assess newborns and mothers for danger signs and breastfeeding assessment included maintaining hand hygiene before examining the newborn, and examination of the newborn for ten danger signs adopted from the 2009 National Newborn Health Strategy [35] as described previously [31, 36–40]. CHWs examined five babies under the supervision of a neonatologist and study physician and received feedback on their performance. (Fig. 1). CHWs also made lactation assessments under the supervision of physicians.

##### Testing for hyperbilirubinemia

CHWs were trained to test newborns for hyperbilirubinemia with a transcutaneous bilimeter, and to determine when a newborn is eligible for home phototherapy based on the birth weight, gestational age, presence of danger signs, and bilirubin level with the aid of an mHealth algorithm, which has been elaborated in Foote et al. [31].

##### Administration of phototherapy

During the training at Dhaka Children’s Hospital and in the field, CHWs were taught how to operate the phototherapy device, to educate parents on how the newborn should be treated with phototherapy in their absence, and to call the study physician if they needed help [31]. CHWs were trained to check the irradiance of the phototherapy device before each use in the home using the manufacturer recommended irradiance meter (Lightmeter V7.0) to ensure that the irradiance is at least 50 µW/cm^2^/nm [31].

###### CHW checklists

**Fig. 2:**
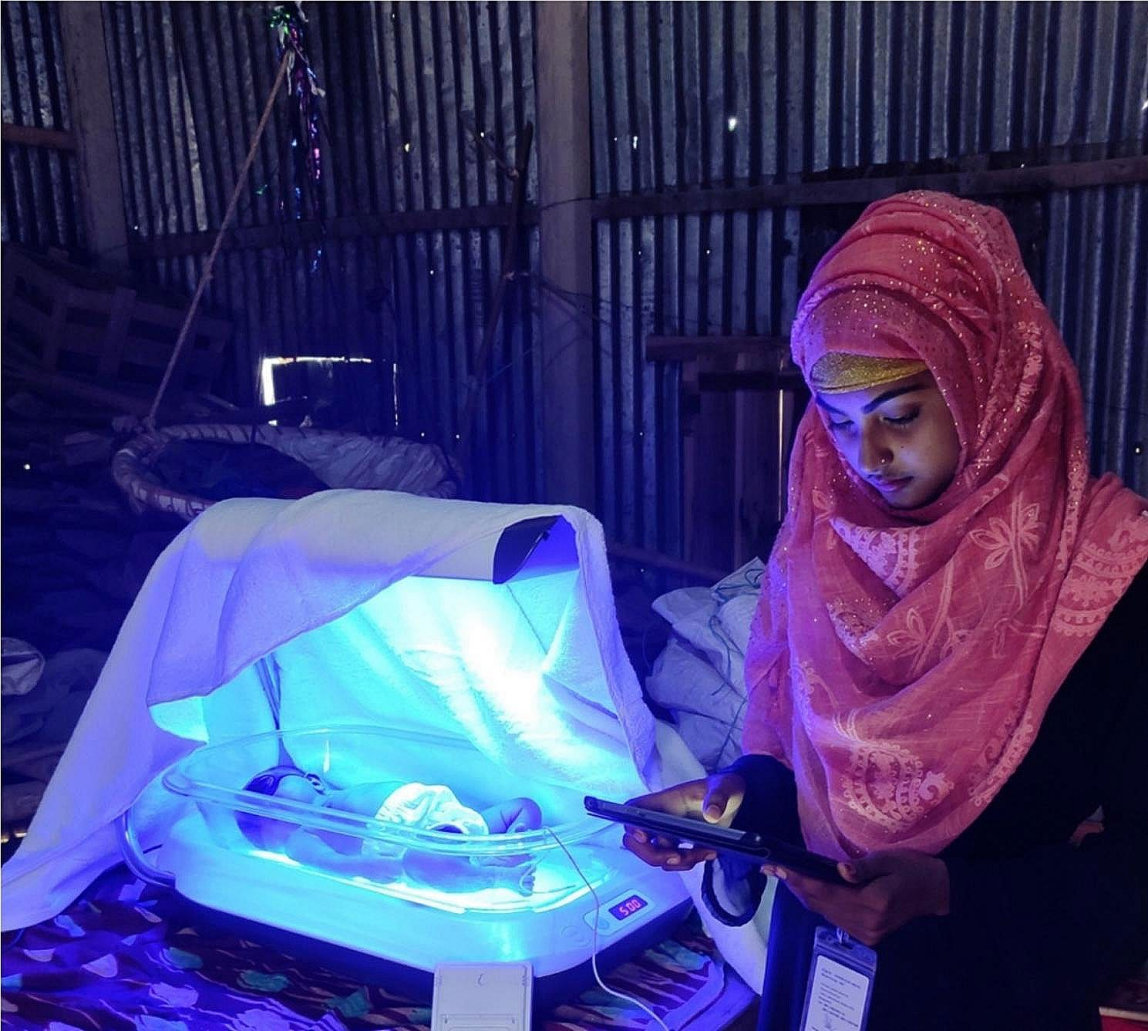
Community health worker monitoring home phototherapy in household in Sakhipur, Bangladesh

CHWs were trained to complete operational and educational checklists with parents and caregivers prior to initiating phototherapy [31]. The operational checklist was completed at each followup visit [31]. The operational checklist was designed to ensure that the measured temperature was between 25 and 27 °C within the bassinet at each CHW visit, that the bassinet is covered by a white blanket (Fig. 2) and is placed in a safe place on a flat surface, that the phototherapy device has a power source and will turn on, and that the infant is in a bassinet face-up with eye-cover and diaper and no other clothes on. The checklist also prompted review of the parental log on daily number of breastfeedings, urination, and defecation, and that the phototherapy unit was turned off overnight from 0000 to 0600. The educational checklist was designed to ensure parents are educated on certain topics related to the phototherapy treatment. CHWs were instructed to explain the need to treat the newborn with phototherapy for neonatal hyperbilirubinemia and the treatment protocol [31]. CHWs are prompted to explain that newborns were to be treated for 2 days with phototherapy with two 6-hours breaks at night from midnight to 6 am, for approximately 36 h of phototherapy treatment. Mothers should take newborns out from under the phototherapy lights for breastfeeding every 2 to 3 h for approximately 20 to 30 min.

After the initial visit, the CHW returned in 4 to 6 h and then daily until phototherapy treatment and post-treatment monitoring was completed. At each visit, CHWs reviewed with families how the phototherapy device works and how to operate the it in the absence of CHWs, and reviewed the operational and educational checklists, including how to clean and dry the bassinet if it gets soiled, monitoring for newborn danger signs, including the importance of contacting the designated CHW if any issue arose, and the need to bring their newborn to the hospital if the phototherapy device is turned off for more than 2 h without resumption of power.

### Prenatal sensitization of mothers and community members on home phototherapy

#### Sensitization of mothers on home phototherapy

CHWs conducted home visits to educate mothers and family members on the signs and symptoms of neonatal hyperbilirubinemia and the possible need for treatment with home or hospital-based phototherapy [31, 41, 42]. Each CHW was assigned 26 to 32 mother-neonate pairs over the implementation period of 4 months. Each mother received at least three sessions starting in their second trimester. CHWs needed to visit 2 to 3 households per day. During visits, CHWs counselled mothers on pregnancy care, safe delivery, essential newborn care, early initiation of breastfeeding, and sensitized the mothers about the signs and symptoms of hyperbilirubinemia and the importance of testing and treatment to prevent mortality and morbidity [43]. Parents were educated to make a plan for how they would get to the hospital if needed for emergency newborn care and treatment.

#### Community sensitization on home phototherapy

As the phototherapy device was new to family members, the study team conducted community sensitization meetings for the parents and family members of newborns or infants, community leaders, school teachers, and physicians from government healthcare facilities. Participants were informed of the importance and benefits of phototherapy and were shown the phototherapy device and how it works, and educated on the safety of the device and possible adverse effects of home phototherapy.

### CHW postnatal evaluation of newborns

CHWs visited infants either within 48 h of birth for home births or the day after discharge from the hospital for infants born in the hospital by vaginal delivery or by caesarean section. A medical technologist measured the newborns’ and mothers’ blood and rhesus types. CHWs made three home visits for maternal and newborn danger signs assessments (within 24 h of birth in case of home birth and after returning to the home in case of hospital delivery and then daily for 2 days). CHWs measured newborn weight; evaluated for newborn danger signs, breastfeeding, and maternal danger signs; and tested newborns for hyperbilirubinemia with a handheld transcutaneous bilimeter [31]. CHWs were guided by the mHealth algorithm to identify newborns eligible for home phototherapy or if newborns needed to be referred for hospital care [31].

### Eligibility criteria for home phototherapy

The inclusion and exclusion criteria for home phototherapy were based on the protocol from Foote et al. [31]. The inclusion criteria were a transcutaneous bilirubin (TcB) level above the 2004 American Academy of Pediatrics (AAP) phototherapy threshold adjusting for gestational age and presence of rhesus or blood type incompatibility and TcB rising ≥ 0.2 mg/dL/hour [31, 44]. The exclusion criteria for home phototherapy were birthweight < 2000 g, gestational age < 35 weeks, presence of newborn danger signs, presence of maternal danger signs, and TcB > 15 mg/dL. Newborns < 2000 g, < 35 weeks, with clinical danger signs, or with TcB ≥ 15 mg/dL were referred for hospital-level care.

### mHealth decision support for management of hyperbilirubinemia

We developed an mHealth program which guided CHWs in neonatal hyperbilirubinemia management. The application contained pertinent information on the participants, including location, gestational age, blood group, and Rh type of pregnant women and their newborns. The application developed on CommCare calculated an estimated delivery date based on the first day of the last menstrual period [31]. Gestational age was calculated by subtracting the birth date from the first day of the last menstrual period. The program recommended phototherapy at home if there were no newborn danger signs and the TcB level was greater than the 2004 AAP phototherapy threshold accounting for gestational age and the presence of blood type or rhesus incompatibility [31, 44]. Phototherapy was also recommended at home if the bilirubin was rising at a rate ≥ 0.2 mg/dL/hour and no danger signs were present [31]. A 2000 gram cutoff for phototherapy excludes most newborns < 35 weeks completed gestational age from receiving home phototherapy and provides an additional measure of safety if the last menstrual period is incorrect. The newborns who met the inclusion criteria were treated for 2 days with phototherapy with two 6-hour breaks at night for approximately 36 h of phototherapy treatment.

Phototherapy treatment was provided by a double surface portable LED phototherapy device (MTTS Firefly phototherapy device), with 12 h of battery backup. The device had a floor area of 66 cm x 38 cm. The irradiance of the top light of the device was 34.8 µW/cm2/nm, and was 50.4 µW/cm2/nm for the bottom light. The phototherapy device had a fixed arm that was 49.5 cm from the bottom of the unit.

### Monitoring of home phototherapy

CHWs visited newborns receiving hoe phototherapy within approximately 4 to 6 h after initiation of phototherapy and then daily for the next four days (three visits in total during phototherapy, and two after completion, detailed in Foote et al. [41]), to perform a danger sign assessment and breastfeeding assessment, and to see if the phototherapy is ongoing as instructed. Newborns with danger signs were referred for hospital care. For all visits after phototherapy was started, CHWs also completed the operational checklist and a short survey to ensure that the phototherapy device was being used as recommended. CHWs reviewed a parental log to check if the newborns were getting adequate feeding. If CHWs identified issues in the survey or checklist, parents were provided education to address the issues or CHWs referred the newborn to the hospital if indicated. CHWs were instructed to contact the study physician if there were any issues with the phototherapy device that CHWs could not resolve or if the family members were refusing recommended treatment. When contacted, the study physician visited the households, assessed the newborn, and referred them to a hospital if there were any danger signs. If there were any issues with the phototherapy device, the physician replaced that device with another device.

### Post-phototherapy monitoring

After home phototherapy was completed, CHWs visited the newborn daily for 2 days starting the day after phototherapy completion and measured the TcB, and assessed for danger signs. Newborns without danger signs and with a TcB < 15 mg/dL for two consecutive days after treatment and a rate of rise of < 0.2 mg/dL/hour were considered to have been successfully treated for hyperbilirubinemia [31]. If any of the criteria were not met, the newborn was referred to Sakhipur sub-district hospital for further evaluation [31].

### On-site supervision and monitoring of CHWs assessments

The CHWs followed an mHealth program that required them to complete assessments that were pertinent to each visit. To validate the accuracy of CHW assessments, the study physician repeated the same assessments of newborn danger signs independently within 4 h. The physician repeated 20% of the total assessments conducted by each CHW. On each day, the physician collected the list of the households the CHWs visited and conveniently picked households to repeat assessments. The study physician and field supervisors observed CHW assessments of danger signs and phototherapy administration and provided feedback as necessary [36].

### Assessment of acceptability and operational feasibility of home phototherapy

We conducted quantitative and qualitative assessments to evaluate the acceptability and feasibility of home phototherapy. To assess feasibility, we evaluated whether the implementation of home-phototherapy was conveniently achieved, accounting for facilitation and barriers experienced by CHWs and families. Acceptability was defined as whether the mother and other caregivers (grandparents and fathers) found the home phototherapy likeable and operable.

#### Quantitative evaluation

A short quantitative survey was conducted by CHWs during each phototherapy visit to assess the acceptability and operational feasibility of home phototherapy for families. Quantitative data were collected using a CommCare-based program [45]. Descriptive analysis was used for the quantitative survey data.

#### Qualitative evaluation

We conducted qualitative evaluation to understand family perceptions, perceived benefits and barriers on home phototherapy. The sample size of in-depth interviews (IDIs) with mothers and focus group discussions (FGDs) with grandparents were determined on the basis of data saturation, as follows: ten IDIs with all study CHWs to assess the operational feasibility of home phototherapy led by CHWs; 11 IDIs (*n* = 11) with mothers who completed home phototherapy to understand the acceptability and feasibility; three IDIs with mothers who either refused or were unable to initiate recommended home phototherapy to understand reasons for refusal or discontinuation; four focus group discussions (FGDs) with parents and grandparents to assess their perceptions regarding home phototherapy.

#### Qualitative data collection

A team of researchers with several years of qualitative research experience conducted IDIs and FGDs with mothers and grandparents of enrolled newborns. IDIs and FGDs lasted 60 to 90 min and were recorded using digital audio recorders. For each of the FGDs, two to three researchers were engaged; one researcher facilitated the discussion, another acted as co-facilitator and the third researcher took hand-written notes to capture important findings and observations by the researchers. The FGDs were conducted in the backyard of houses and interviews were conducted inside the house to maintain privacy and confidentiality.

#### Qualitative data analysis

We analyzed qualitative data using thematic content analysis techniques following an inductive process. Audio recordings were transcribed verbatim into Bengali in the word processor, translated into English and coded using the qualitative data analysis software Atlas.ti (version 5.2). Translation errors were checked by a second researcher. For coding in Atlas. Ti, the research team prepared a set of themes after reading all the transcripts, summaries, and written notes. Four team members coded each transcript based on our primarily generated themes. During the coding process, the team also identified and included new codes. Once all the codes were identified, we sorted and collated coded data extracts into generated themes.

### Ethical approval and consent to participate

This study was conducted under the ethical principles of the Declaration of Helsinki. Written informed consent was obtained from the all the study participants prior to quantitative surveys, IDIs, FGDs and audio recordings. Participants were offered the opportunity to read the consent form fully and ask and receive answers to any questions before giving their consent. The study protocol was approved by the institutional review boards of icddr,b (IRB/ERC no. PR- 19,004) and Stanford University (protocol 52,625).

## Results

### Demographic characteristics of study participants

A total of 54 individuals took part in the qualitative study, including all study CHWs (*n* = 10), mothers of newborns receiving home phototherapy (*n* = 11), mothers of newborns refusing or unable to complete home phototherapy (*n* = 3), parents (*n* = 16) and grandparents (*n* = 14) of the newborns who were offered phototherapy (Table 1). The mean age of participating mothers in the IDIs (*n* = 14) was 24.5 [standard deviation (SD) 4.2] years, four (28.6%) completed primary school education (5 years of education), and ten (71.4%) completed secondary school (10 years of education). A majority of the mothers were homemakers. The mean age of the parents and grandparents participating in the FGDs was 55 years and 32 years, respectively. The mean age of CHWs was 29 years (SD 5.8); six CHWs completed university-level education (15–16 years of education) and the four CHWs completed higher secondary education (12 years of education).

**Table 1:** Characteristics of study participants enrolled in the evaluation by qualitative method

Characteristics	Focus Group Discussions		In-depth-interviews	
	Grandparents	Parents	Mothers	CHWs
	(*N* = 14)	(*N* = 16)	(*N* = 14)	(*N* = 10)
Mean age, years (standard deviation)	55 (6)	32 (4.8)	24.5(4.2)	29 (5.8)
Average monthly income, BDT	10,000	15,000	16,000	10,000
**Highest level of Education completed**				
Primary (5 years of schooling) education	5 (35%)	5 (32%)	4 (28%)	0 (0%)
Secondary to higher secondary (10–12 years of schooling)	0 (0%)	8 (50%)	10(71%)	4(40%)
Graduation (15–16 years of education)	0 (0%)	3 (18%)	0 (0%)	6 (60%)
No institutional education	9 (65%)	0 (0%)	0 (0%)	0 (0%)
**Profession**				
Housewife	9 (65%)	9 (56%)	11(78%)	0 (0%)
Agriculture/Day labor in agricultural work/household	4 (28%)	3 (18%)	3 (22%)	0 (0%)
Government Service	0 (0%)	2 (12%)	0 (0%)	0 (0%)
Private service	0 (0%)	2 (12%)	0 (0%)	10 (100%)
Retired/no job	1 (7%)	0 (0%)	0 (0%)	0 (0%)
**Sex**				
Male	4 (28%)	7 (44%)	0 (0%)	0 (0%)
Female	10 (72%)	9 (56%)	14(100%)	10 (100%)

### Quantitative survey of CHW visits with mothers during home phototherapy

During 93% of all CHW visits (56 of 60 visits), they found on arrival that the phototherapy machines were turned on with the newborn inside the bassinet. (Table 2). All the newborns had eyepatches while being treated with phototherapy and in one instance an infant was wearing clothes during phototherapy treatment. When asked about the reason for putting clothes on, the mother responded that she thought the infant was cold; the CHW then provided instructions to not clothe the baby during phototherapy. At all visits, the temperature inside the phototherapy device was at the 25 to 27 °C target during phototherapy. During 3% of CHW visits (2 of 60 visits), mothers reported problems while continuing phototherapy; in one case the infant was cold and in the other case the infant was crying continuously. The phototherapy unit’s 12-hour battery backup prevented power loss in 10/11 instances where there was household power loss as household power was restored before the battery ran out of power. In the household where there was a prolonged power loss and home phototherapy could not be initiated, the newborn was referred for hospital care. During 7% of CHW visits (4 of 40), mothers reported turning off the device for a few minutes when newborns were irritated and continuously crying. During nearly all (95%, 57 of 60) of the visits, mothers could mention at least one newborn danger sign (Table 2).

**Table 2:** CHWs checklist and survey of mothers during newborn receiving phototherapy treatment

	Question	4–6 h after phototherapy initiation n (%)	1 day after phototherapy initiation n (%)	2 days after phototherapy initiation n (%)
CHW checklist during phototherapy	Was the phototherapy unit on at arrival? (Yes)	18 (90)	18 (90)	20 (100)
	Is the temperature inside the bassinet between 25–27 °C?	20 (100)	20 (100)	20 (100)
	Is the device is placed on a flat surface? (Yes)	20 (100)	20 (100)	20 (100)
	Does the infant have clothes on while under phototherapy? (Yes)?	0	1 (6)	0
	Is the infant faced down (Yes)?	1 (6)	0	0
	Is the baby wearing eye patches while under phototherapy unit (Yes)?	18 (100)	18 (100)	20 (100)
Survey with mothers	Any reported problem during phototherapy?	0	1 (5)	1 (5)
	The type of problems faced-			
	Infant continuously cried	0	0	1 (100)
	Infant too cold	0	1 (100)	0
	Number of events of household power loss during phototherapy	2	5	4
	Number of events where the phototherapy service interrupted due to household power loss	0	1	0
	Was the phototherapy device on except night?	18 (90)	19 (95)	19 (95)
	Reason for turning the device off?- The baby was irritated	2 (100)	1 (100)	1 (100)
	How long was the device turned off? Average (minutes)	30	20	20
	How many times in 24 h should the newborn be breastfed during phototherapy? (Mean, Min, Max) (Ranges)	11, 10, 12	12, 10, 14	12, 10, 14
	For approximately how long was the infant out of lights for feeding? Minutes (Mean)	15	17	12
	Number and percentage of mothers that could name at least one danger signs	19 (95)	19 (95)	20 (100)
	Number and percentage of mothers that knew to call the CHW or study physician if there was problem	19 (95)	20 (100)	20 (100)

### Acceptability of home phototherapy: mothers’ and grandparents’ perspectives

#### Effective treatment method for infants with jaundice

Out of 23 newborns recommended home phototherapy, 20 (86%) families completed treatment, two families refused to initiate treatment (8%) and one (4.3%) family was unable to initiate treatment due to inadequate household power supply. Nearly all the mothers whose newborn was treated with home phototherapy mentioned that home phototherapy is an effective method for treating newborn jaundice. They reported that their baby improved dramatically after receiving the therapy. They noticed that the yellowish coloration of the skin reduced, the feeding time increased, and newborns seemed more playful. The mothers also stated that because they were sensitized about the signs and symptoms of jaundice, they understood that their child could become sick and in need of phototherapy treatment.

One mother aged 33 years mentioned, *“My child’s eye was yellow in color and he was not breastfeeding very well, so I thought the baby was having trouble because of jaundice. When the apa (CHW) advised home phototherapy, I agreed immediately. I think the treatment was very effective for my child and he is growing well now”*.

#### Home phototherapy was convenient and cost-effective

The majority of the mothers and grandparents mentioned that they appreciated the services provided by the CHWs. They reported that their families usually did not visit doctors for neonatal jaundice as long as the baby is not severely sick. However, after sensitization of the community on neonatal jaundice through community meetings and prenatal educational sessions, they had knowledge about the signs, symptoms, treatment, and complications of neonatal jaundice. But still, they believed that visiting hospitals for neonatal jaundice is not a good choice for them as this cost a lot of time and money and they are happy that their babies received home phototherapy. They also liked this service, as all the family members can look after the babies at home and the mother can take rest whenever needed. On the other hand, hospital phototherapy is expensive as they had to stay at the hospital, which results in the loss of daily wages of the family members accompanying the baby.

One grandparent reported, *“We people in Sakhipur prefer not to visit government hospitals as it is overcrowded and the number of doctors and nurses are less than required. It is also true that the doctors and nurses are too busy that they could not provide enough time to individual patients”*.

#### Simple operation of the phototherapy device

During IDIs, the mothers stated that the device was new to them but they quickly learned to operate the machine. They mentioned that CHWs brought the machine to households and set up the machine. Then they trained the mothers and other caregivers to operate the machine, specifically how to turn the machine on and off. During FGDs, the grandparents and fathers mentioned that CHWs educated them on how to clean the bassinet if it got soiled during phototherapy.

One mother reported, *“We kept the baby continuously in the machine, and breastfed the baby every 2 hours. When my child defecated in the machine, I took out him, cleaned him, and then again put him in the machine. During nighttime, I and my sister-in-law operated the machine as CHW apa (sister) was not present at night. We found it quite simple to operate”*.

#### Maintaining the nutrition of mothers and babies is convenient at home

Mothers and grandparents mentioned during IDIs and FGDs that they could properly feed mothers during home phototherapy. They added that CHWs sensitized them about the importance of early initiation and continuation of breastfeeding. They said it would be difficult to maintain the nutrition of the mother while breastfeeding if she and the baby were at the hospital. The mothers found that their daily life was unhampered when their babies got home phototherapy. Mothers mentioned that they could easily breastfeed their baby during home phototherapy, while at a hospital there is little privacy to breastfeed the baby as the rooms are shared by many patients.

### Operational feasibility of CHW-led home-based neonatal hyperbilirubinemia management

#### Facilitators of home phototherapy

##### CHW training in neonatal hyperbilirubinemia management

All the CHWs felt that the hands-on training on newborn hyperbilirubinemia management made them confident to handle newborns. They reported that they were well prepared to assess newborns for danger signs and suggest management accordingly. One CHW aged 30 years reported, *“I received training in neonatal jaundice, types of neonatal jaundice, and how to take care of the newborn. I also learned how to screen the baby with the handheld device and how to operate the phototherapy device”*.

Another CHW reported, *“We have directly seen the newborn babies with complications at Dhaka Children’s hospital. We saw some babies were very sick. After the hands-on training, we were confident that we could assess the danger signs and manage phototherapy at home”*.

The CHWs mentioned that the 5 days training package was designed in such a way that they could easily learn and practice at the same time.

##### CHW work load

CHWs felt that because they were assigned a particular number (*n* = 26 to 32) of households they could manage their work efficiently. They also mentioned that because they had multiple visits with the same clients, they had the opportunity to develop a bond with the pregnant mothers and their family members which was crucial to convincing the family members when home phototherapy was needed.

One CHW aged 32 years stated, *“We came to the project office every morning and then started visiting households at 9am. Most of the households are within 30–60 minutes’ distance by local transport. We visited 2–3 households every day. We finished assessments before 4pm, because if we refer any baby or mother after 4pm it’s difficult for them to go to the hospital”*.

##### mHealth program provided novel job aids

The mHealth program was praised by all the CHWs, who reported that it was a comprehensive program that helped them to identify the mothers, and provided their household location including their blood group and the infant’s height, weight, and risk factors for developing neonatal hyperbilirubinemia. CHWs stated that the mHealth program made their job easy as it assisted them in decision-making regarding the need for phototherapy. They also stated that there was no chance to skip or forget any steps in the assessment of newborn danger signs while continuing phototherapy. They also reported that the mHealth program was very simple, easy to operate, and had minimal chance of error.

One CHW aged 25 years quoted, *“I was very comfortable using the program on a tablet though I did not have any previous experience of using such a program. Once I input the mother’s ID it shows me the information of mothers and babies. Once I input the birthweight, blood group, danger signs assessment results, and TcB of the baby, the program showed whether this baby will receive home phototherapy, hospital phototherapy or will be referred to hospital for any other danger signs”*.

Another CHW aged 32 years stated, *“When I completed the assessment and saved the input, it provided a full picture of the mother and baby. I felt very proud when I could refer the baby or start phototherapy at home.”p*.

##### CHW perception of parental confidence in the provision of home phototherapy

The CHWs reported that the mothers were compliant during home phototherapy. When the mothers were told that their babies need home phototherapy, most of them immediately agreed. CHWs also mentioned that the physician’s visit (repeat assessments and visits to three families at their request) increased the confidence of the parents in the phototherapy decision. The mothers were keen to understand the operations of the phototherapy machine. CHWs reported that most of the mothers and other caregivers learned the operation of the phototherapy machine with minimal instruction. The caregivers also documented the breastfeeding, defecation, and urination frequency of newborns in the provided logger.

One CHW aged 36 years (FGD 1) stated, *“Parents were always supportive of home phototherapy as it did not impose extra workload on the family members, most of the time others can take care of the baby. They did not have to travel and stay at hospital, so they can continue their household chores and other business”*.

#### Operational challenges for CHWs for home phototherapy

CHWs were faced with a number of challenges to implement home phototherapy which included transportation to remote areas, instilling confidence in family members in the safety of the device, electrical outrages and gaining the trust of family members to have the CHW treat newborns with phototherapy in the home.

##### Capacity building of mothers with limited education and family members on operating the phototherapy device

A few mothers and family members were hesitant to use a new device. Most of the mothers overcame this hesitancy in the prenatal phase as a result of CHW prenatal visits and demonstration of the phototherapy device, including its components and how it works and education on potential adverse effects. All mothers and families received additional counseling in the postnatal period by the CHWs if phototherapy was recommended at home or in the hospital. Mothers also had the opportunity to request to speak to the study physician, who provided education to mothers.

##### Transportation of the phototherapy device

The CHWs reported that transporting the phototherapy machine to some of the households was difficult for them. They struggled to find a vehicle to carry the machine to households located in remote areas. They mentioned that some of the villages are remote, up steep roads that are muddy and slippery in the monsoon season. It was difficult to carry the phototherapy device, which weighted 13 kg, into the hard-to-reach villages. Later in the project, a van was hired by the project to carry the device to the households where home phototherapy was required. They also mentioned that they tried to complete household visits before dusk as some of the households are far and they had to return home before dark.

##### Family members’ fear of a new device

The mothers who refused home phototherapy for their newborns mentioned that their family members were afraid to put their baby into the phototherapy basinet. Even though they were sensitized during the prenatal period about the phototherapy machine, including its usage and safety, a hesitancy was still there when the baby was advised phototherapy. CHWs reported that they overcame the situation with additional counseling.

##### Interruption in electric supply

Electrical outages are very common at sub-district level in Bangladesh. The CHWs said that often there was a power cut while newborns were receiving phototherapy, however, phototherapy continued uninterrupted as the phototherapy device had a 12-.

hour battery back-up. The phototherapy unit’s 12-hour battery backup prevented power loss in 10/11 instances where there was household power loss during administration of home phototherapy during the study period. In 10/11 instances, household power was restored before the battery ran out of power. In the household where there was a prolonged power loss and home phototherapy could not be initiated, the newborn was referred for hospital care.

##### Lack of confidence in CHW skills

CHWs reported that given the level of intervention which seemed clinical and demanded specific skills, three families doubted whether CHWs should conduct these activities. Those families requested verification from a physician that their babies needed phototherapy. The study physician’s visits increased the confidence of those families in the decision provided by CHWs and resulted in completion of home phototherapy.

### Refusal or discontinuation of home phototherapy: perspective from mother,grandparents and CHWs

Two families refused to initiate recommended home phototherapy. One family did not trust in the CHW’s ability to successfully initiate home phototherapy. The CHW stated that family members doubted in the CHWs skills in operating the phototherapy device which led to the family refusing to continue phototherapy. This newborn was referred to the hospital for care. Another family was not comfortable with the phototherapy device. They said the baby was uncomfortable and continuously crying after putting the infant under the device and that concerned them. Inadequate power supply was a reason for not being able to initiate phototherapy in one of the houses (Table 3). Electrical outages are common at the sub-district level in Bangladesh. Despite instances of household power loss, phototherapy continued uninterrupted in all but one instance due to the 12-hour battery backup in the phototherapy unit. CHWs mentioned that household electicity was more of a concern in winter, as they had to provide a room heater to maintain the room temperature between 25 to 27ºC. “*At one house the electric line was short-circuited after connecting the room heater. The family members got panicked in that situation.*” This family was unable to initiate recommended home photototherapy due to inadequate household power supply and the newborn was referred for hospital care (Table 3).

**Table 3:** Overview of key reasons for refusal or discontinuation of home phototherapy by three families

Reasons identified	Important Quotes
Lack of trust in CHWs	Mother of newborn mentioned *“When CHW was setting up all the devices, I found that she did not know the right procedure so she called someone and asked about the rules of setting the machine. I felt a little doubt that whether she knew how to use these devices, but I believed that she would do what was safe. But then child started crying and the situation worsened. I lost my belief in her and I told her to stop doing that. My family members were totally disappointed with the situation. They told me to immediately stop using this device and take the baby to the doctor.”*
Uncomfortable with the new device	The mother mentioned, “*The baby cried continuously after putting under blue light. The grandfather of the baby was afraid, that the baby was not feeling well in this device and asked CHWs to stop phototherapy”*.
Loss of household power	The mother reported, “*CHW did not tell us that the machine run with electricity*. *When they connected the room heater our electricity line got burnt because of a huge load from the machine. So, we stopped phototherapy*.” CHW (29 years) assigned to that household mentioned, *“At one house the electric line was short-circuited after connecting the room heater. The family members got panicked in that situation.*”

## Discussion

CHW-led home phototherapy was acceptable and feasible for caregivers in rural Bangladesh. Mothers liked home phototherapy as the device was easy to use, effective, did not interfere with breastfeeding, and avoided a hospital visit saving them time and money. CHWs found that home phototherapy was operationally feasible and noted that intensive training, an mHealth aid which supported them in decision-making processes, prenatal sensitization leading to trust-building with families, and the availability of a physician for questions and support were facilitating factors. The findings of this work are similar to studies conducted in middle- to high-income countries including Sweden, Iran and the USA where home phototherapy was found to be a feasible alternative to hospital phototherapy for otherwise healthy newborns with hyperbilirubinemia if daily visits by health workers and 24/7 telephone support can be provided [11, 12, 46, 47].

CHWs felt confident in managing newborns with hyperbilirubinemia as they received intensive hands-on training on newborn assessment and management. Their assessments of danger signs and decision-making for home phototherapy or referral were facilitated by an mHealth platform which was designed to limit mistakes, prevent assessments from being missed, and facilitated treatment and referral decisions. Other studies showed mHealth to be an effective tool in home-based identification of neonatal illness and in supporting clinical decision-making [48–51]. A nested study conducted under this trial showed that CHW identification of neonatal danger signs aided by mHealth showed moderate to high validity (sensitivity 93.3% and specificity 96.2%) in comparison to physician assessment [34]. mHealth platforms may reduce CHW training requirements while maintaining performance. Gaining trust with family members through prenatal sensitization on home phototherapy facilitated acceptance and successful completion of home phototherapy. Other studies conducted in LMICs found that hands-on training and community sensitization facilitated successful implementation of home-based perinatal care interventions, and diabetes and hypertension management [52–54].

One barrier found in this study was capacity development of mothers on operation of the device and in understanding the need for postnatal testing and in some cases treatment for neonatal hyperbilirubinemia. Our study had a sensitization and capacity development program that occurred during prenatal and postnatal CHW visits. Our qualitative data suggests that sensitization helped improve acceptability [43]. Sensitization of mothers will be a key feature to enable successful implementation in low resource settings where postnatal care is limited and there is not familiarity with neonatal hyperbilirubinemia diagnosis or treatment. A key barrier faced by CHWs in implementing home phototherapy was providing services to remote areas due to limited transportation. Carrying phototherapy devices to remote villages was difficult for the CHWs. However, they overcame this barrier by arranging specific transport for phototherapy devices. Transportation is often an important barrier to providing health services in remote areas of low-income countries [55–57]. Remote, rural areas are often where neonatal morbidity and mortality are highest due in part to a lack of access to quality healthcare, highlighting the importance of developing the ability for CHWs to provide high quality household-level care in remote settings [58–62].

Variable home electrical service was a barrier from the perspectives of both CHWs and caregivers. This finding correspondents with other studies conducted in similar settings where limited access and interrupted electricity service was a barrier for implementing home-based mHealth services [63–65]. The 12-hour phototherapy battery back-up power prevented interuptions in phototherapy treatment in 10 of 11 instances of power loss during treatment and enabled phototherapy to continue uninterrupted. In one instance, inadequate household power supply prevented a newborn from receiving home treatment and this newborn was referred for hospital care. Having a reliable power supply is essential for providing successful household treatment and the need for reliable electricity may limit the ability to provide home phototherapy in some LMIC settings.

Another important barrier in initiating home phototherapy was the lack of confidence of mothers and family members in CHWs’ skills in providing phototherapy to their newborns. CHWs’ skill and competency to provide curative services at the community level are often doubted by mothers and community members, an important barrier in community-based curative intervention implementation [66, 67]. In our study, this was overcome through prenatal sensitization, the use of a point-of-care instant diagnostic device (transcutaneous bilimeter), and having access to a study physician to answer parental questions [43]. The objective measurement from the transcutaneous bilimeter helped improve confidence from families that phototherapy was needed [42]. The refusal rate for home phototherapy was 13% (3 of 23 families), which was low compared to other CHW-led home-based curative treatment approaches where refusal rates of home care for very severe disease and possible very severe disease were 23% and 38%, respectively [28].

The 2004 AAP neonatal hyperbilirubinemia management guidelines, including phototherapy treatment thresholds, were used in this study [44]. An updated AAP guideline was published in 2022, after this study was initiated, which increased the bilirubin thresholds for phototherapy treatment by 0-2 mg/dL [44, 47]. In the 2022 guidelines, home phototherapy treatment could be considered at a bilirubin level between 2 mg/dL below and 1 mg/dL above the 2022 hospital-level bilirubin treatment thresholds. While some LMICs use bilirubin thresholds for phototherapy treatment that are less than both the 2004 and 2022 guidelines, the phototherapy treatment thresholds used in this study are similar to the 2022 guideline’s home phototherapy thresholds [44, 47, 68, 69]. The WHO guidelines recommend a phototherapy treatment threshold for bilirubin values above 10–15 mg/dL [70]. The choice of phototherapy threshold likely did not affect the main conclusions of the study related to operational feasibility and acceptability amongst parents and CHWs.

Our study had some limitations. Our sample of 23 newborns for whom home phototherapy was recommended was limited. This paper details the operational feasibility and qualitative acceptability of home treatment amongst families that received home phototherapy and the CHWs that provided phototherapy and did not evaluate the effectiveness of home phototherapy on clinical measures. The sample size is too small to understand all potential adverse effects and operational barriers that could occur with CHW-administered home phototherapy in households in Sakhipur, Bangladesh. Further studies in a variety of LMICs settings should enroll a larger sample size to better understand clinical outcomes from phototherapy treatment for neonatal hyperbilirubinemia in LMICs and potential adverse effects and operational barriers. The number of IDIs and FGDs were limited, but determined on the basis of data saturation. This paper did not include parents whose newborns were referred for hospital phototherapy. Equipping health care facilities at resource constrained settings with adequate resources for phototherapy treatment and capacity development of health care workers will play a crucial role to ensure successful referral and treatment. The CHW workload in this study may have been less than that of government CHWs. In government-provided CHW care, one health worker is responsible to provide family planning services to 750 eligible couples and 200 subsequently pregnant women. Utilizing government recruited CHWs with adequate incentive for every successful diagnosis and treatment of neonatal hyperbilirubinemia can be an alternative solution in resource constrained settings [71, 72]. Further studies can focus on the utilization of government CHWs for home based care, estimation of patient load and evaluative feasibility of alternative incentive method in the context of a larger program. The capital cost of phototherapy device that we used in our study was high (USD 1400); however, after the initial purchase, ongoing cost to charge the device is minimal. The cost of phototherapy device may decrease overtime as well. Further studies on the cost implications and alternative approaches can determine the feasibility of large scale use.

We used a portable phototherapy device that has a floor area of 66 cm x 38 cm which was recommended to be placed on a flat surface inside the home. All families in the study that needed phototherapy had a flat surface to put the device in their home. Having a flat surface on which to place the phototherapy device may be a challenge in some settings where household indoor space is limited, which could limit the widespread use of this solution.

## Conclusions

Home-based phototherapy for neonatal hyperbilirubinemia management in rural Bangladesh was operationally feasible and acceptable for families and CHWs. Factors that increased acceptability of home phototherapy included prenatal sensitization, simple operation of the phototherapy device, point-of-care diagnosis, effectiveness in improving neonatal jaundice symptoms, facilitation of breastfeeding while treatment occurred, savings of time and costs, and avoidance of hospital visits. CHW hands-on training on neonatal hyperbilirubinemia management and an mHealth application that guided CHWs through the diagnosis and treatment protocol were some of the key factors mentioned by CHWs as important for successful implementation. Refusal of home phototherapy was limited. The main causes of home phototherapy refusal were parental lack of trust in CHWs’ skills, loss of household power, and fear of the phototherapy device. These findings can inform future implementation of home phototherapy to treat neonatal hyperbilirubinemia in LMICs. Further research should explore how a CHW-led neonatal hyperbilirubinemia treatment program could be integrated into care delivery systems in LMICs.

## Data Availability

Institutional Review Board approval was obtained for public sharing and presentation of data on a group level only. To maintain participants’ anonymity and confidentiality, the data set generated during the study will not be publicly available but is available from the corresponding author on reasonable request.

## Abbreviations

CHW: Community Health Worker
LMIC: Low- and middle-income countries
LED: Light Emitting Diode
IDI: In-Depth Interview
FGD: Focus Group Discussion
AAP: American Academy of Pediatrics
TcB: Transcutaneous Bilirubin
ANC: Antenatal Care
